# Dietary restriction and gonadal signaling differentially regulate post‐development quality control functions in *Caenorhabditis elegans*

**DOI:** 10.1111/acel.12891

**Published:** 2019-01-15

**Authors:** Nufar Shpigel, Netta Shemesh, Mor Kishner, Anat Ben‐Zvi

**Affiliations:** ^1^ Department of Life Sciences, The National Institute for Biotechnology in the Negev Ben‐Gurion University of the Negev Beer Sheva Israel

**Keywords:** aging, *daf‐16*, dietary restriction, germline stem cells, *pqm‐1*, proteostasis, quality control

## Abstract

Protein homeostasis is remodeled early in *Caenorhabditis elegans* adulthood, resulting in a sharp decline in folding capacity and reduced ability to cope with chronic and acute stress. Endocrine signals from the reproductive system can ameliorate this proteostatic collapse and reshape the quality control network. Given that environmental conditions, such as food availability, impact reproductive success, we asked whether conditions of dietary restriction (DR) can also reverse the decline in quality control function at the transition to adulthood, and if so, whether gonadal signaling and dietary signaling remodel the quality control network in a similar or different manner. For this, we employed the *eat‐2* genetic model and bacterial deprivation protocol. We found that animals under DR maintained heat shock response activation and high protein folding capacity during adulthood. However, while gonadal signaling required DAF‐16, DR‐associated rescue of quality control functions required the antagonistic transcription factor, PQM‐1. Bioinformatic analyses supported a role for DAF‐16 in acute stress responses and a role for PQM‐1 in cellular maintenance and chronic stress. Comparing the stress activation and folding capacities of dietary‐ and gonadal‐signaling mutant animals confirmed this prediction and demonstrated that each differentially impacts cellular quality control capabilities. These data suggest that the functional mode of cellular quality control networks can be differentially remodeled, affecting an organism's ability to respond to acute and chronic stresses during adulthood.

## 1 INTRODUCTION

Cells cope with protein damage by employing quality control machineries, such as molecular chaperones, the ubiquitin–proteasome system (UPS), or the autophagy machinery (Bar‐Lavan, Shemesh, & Ben‐Zvi, 2016; Bett, 2016; Jackson & Hewitt, 2016). These basal machineries can be supported by the activation of stress response pathways, when damaged proteins accumulate (Sala, Bott, & Morimoto, 2017). While the expression of quality control machineries can vary between tissues, such systems are also regulated cell non‐autonomously by intertissue signaling (O'Brien & van Oosten‐Hawle, 2016), resulting in an overall remodeling of quality control network composition and the activation of acute or chronic stress responses. Because the specific composition of folding and degradation machineries can impact network specificity and the capacity to repair or remove damaged proteins (Bar‐Lavan, Shemesh, Dror, et al., 2016), it is important to understand the dynamics of the quality control network in the face of different challenges.

Remodeling protein homeostasis (proteostasis) can be mediated by endocrine signals released from neurons or the reproductive system (Sala et al., 2017; Shai, Shemesh, & Ben‐Zvi, 2014). A well‐studied example of proteostasis remodeling is the early switch in the regulation of quality control networks seen at the onset of *Caenorhabditis elegans* reproduction, associated with a reduced folding capacity and stress response activation (Labbadia & Morimoto, 2015; Shemesh, Shai, & Ben‐Zvi, 2013). Inhibited activity of steroid hormone signaling at the transition to reproductive adulthood results in a sharp decline in both folding capacity and stress activation, whereby the functions of various transcription factors regulating the expression of quality control machineries, including HSF‐1/HSF1, DAF‐16/FoxO, PHA‐4/FoxA, and SKN‐1/Nrf2, are modulated (Berman & Kenyon, 2006; Lapierre, Gelino, Melendez, & Hansen, 2011; Shemesh et al., 2013; Steinbaugh et al., 2015). This is associated with age‐dependent misfolding and the aggregation of various metastable proteins and a general decline in healthspan (Ben‐Zvi, Miller, & Morimoto, 2009; Chang, Kumsta, Hellman, Adams, & Hansen, 2017; David et al., 2010; Shai et al., 2014; Vilchez et al., 2012).

Endocrine signaling pathways, such as the insulin/IGF‐1 signaling (IIS) pathway or the gonadal longevity pathway, and environmental signals, such as temperature and nutrient availability, are often associated with lifespan and healthspan determination (Riera, Merkwirth, Magalhaes Filho, & Dillin, 2016; Sala et al., 2017). These signals are mediated by differential regulation of a wide range of transcription factors, some of which control the expression of various quality control machineries. For example, germline stem cell (GSC) arrest modulates the transcriptional reprogramming of HSF‐1, DAF‐16, PHA‐4, and SKN‐1 and thus counters the decline in folding capacity and stress activation (Berman & Kenyon, 2006; Chang et al., 2017; Labbadia & Morimoto, 2015; Shemesh, Meshnik, Shpigel, & Ben‐Zvi, 2017; Shemesh et al., 2013; Steinbaugh et al., 2015). Given how the remodeling of quality control networks converges on a similar set of transcription factors, we asked whether different endocrine or environmental signals result in equivalent or distinctive quality control networks.

Dietary restriction (DR) is the most robust intervention that increases the lifespan and healthspan of many organisms (Fontana & Partridge, 2015). In *C. elegans*, different DR protocols were shown to extend lifespan, as well as to repress proteotoxicity, modulate stress response activation, and improve proteostasis (Kapahi, Kaeberlein, & Hansen, 2016). We, therefore, asked whether DR can modulate the proteostasis switch seen at the onset of reproduction and, if so, how DR affects somatic quality control networks in comparison with their GSC‐dependent reprogramming.

## 2 RESULTS

### 2.1 DR restores heat shock response activation during adulthood

**Figure 1:**
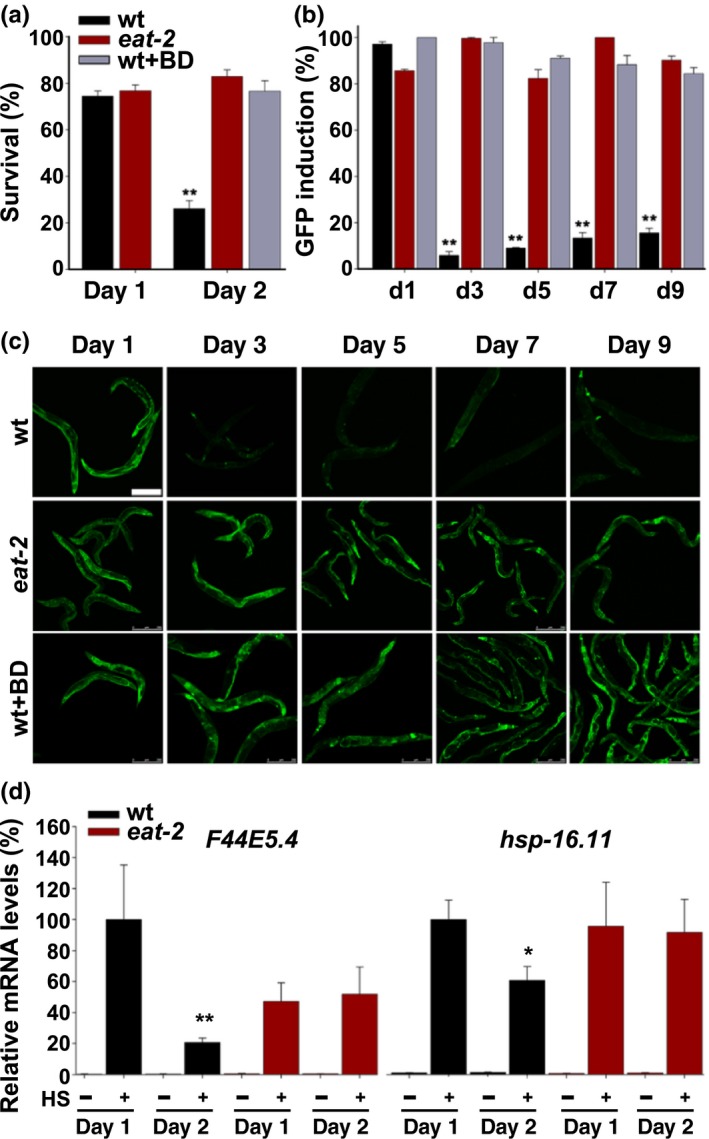
DR modulates HS repression at the transition to adulthood. (a) Survival rates of age‐synchronized wt or *eat‐2(ad453)* animals fed ad libitum or BD‐treated for 24 hr. Animals were subjected to HS (6 hr at 37°C) on day 1 or day 2 of adulthood, and survival was assayed. (b–c) HS‐regulated GFP_HS_ expression. Age‐synchronized GFP_HS_ or *eat‐2(ad453);*GFP_HS_ animals fed ad libitum or BD‐treated for 24 hr prior to stress were imaged following a short HS (90 min at 37°C). The percent of animals showing GFP in the gut was scored. Scale bar is 250 µm. (d) Expression levels of HS genes. mRNA levels of *F44E5.4* and *hsp‐16.11* from age‐synchronized wt or *eat‐2(ad453)* animals untreated or subjected to HS (90 min at 37°C). Data presented are normalized to those from day 1 HS‐treated wt animals. *p *values were calculated by comparison with day 1 adults of the same strain. (*) denotes *p* < 0.05, and (**) denotes *p* < 0.01

To examine whether DR can modulate the proteostatic switch at the transition to reproductive adulthood, we employed two different DR regimens: (a) a genetic model utilizing *eat‐2* mutant animals that lack a specific nicotinic acetylcholine receptor subunit required for pharyngeal pumping (McKay, Raizen, Gottschalk, Schafer, & Avery, 2004) and (b) dietary deprivation (DD) that corresponds to a complete removal of food post‐development (Steinkraus et al., 2008). Because proteomic changes in response to starvation are completed within hours (Larance et al., 2015), removal from food, defined here as bacterial deprivation (BD), was applied for a short period of 24 hr prior to scoring. The survival rates of wild‐type (wt) animals first challenged by heat shock (HS) after the onset of reproduction (6 hr at 37°C; day 2) were strongly reduced, as compared to the survival rates of wt animals first challenged as young adults (day 1; 26.1 ± 3% and 74.5 ± 2%, respectively; Figure 1a). In contrast, the survival rates of *eat‐2(ad453) (eat‐2)* mutant animals challenged by HS did not decline significantly until day 9 of adulthood (77 ± 2, 82 ± 4% and 69 ± 6%, on day 1, 2, and 9 of adulthood, respectively; Figure 1a and Supporting Information Figure S1a). A rescue of HS survival rates was observed under different cultivation temperatures, as well as using different *eat‐2* alleles. Animals harboring either the *ad453* or *ad1116 eat‐2 *mutant allele, grown at 15°C, maintained high survival rates, as compared to wt animals (79 ± 3%, 86 ± 2%, and 36 ± 6%, respectively), when challenged by HS (4 hr at 34°C) on day 2 of adulthood (Supporting Information Figure S1b). Likewise, the survival rates of wt animals removed from food for 24 hr (BD‐treated) remained high when challenged by HS (6 hr at 37°C) on day two of adulthood (77 ± 4%; Figure 1a). Thus, DR resulted in the maintenance of high HS survival rates during *C. elegans* adulthood.

To examine the activation of the HS response directly, we next monitored wt and *eat‐2* animals carrying a transcriptional reporter in which an *hsp‐16.2* promoter regulates green fluorescent protein (GFP_HS_) expression patterns. While GFP_HS_ was induced in various somatic tissues in both wt and *eat‐2 *day 1 adults (97 ± 1% and 86 ± 1%, respectively), on day 3 of adulthood, strong GFP_HS_ expression was only detected in *eat‐2* animals (Figure 1b and c). *eat‐2* mutant animals maintained their ability to induce GFP_HS_ in somatic tissues at least up to day 9 of adulthood (90 ± 2%). GFP_HS_ induction was also observed following a 24 hr BD treatment of GFP_HS_ animals prior to HS (98 ± 2%; Figure 1b and c), similar to DD (Steinkraus et al., 2008). To complement this approach, we compared the induction of representative HS genes by monitoring changes in mRNA levels following HS on days 1 or 2 of adulthood, using qPCR. While the induction of *Hsp70* (*hsp‐70* and *F44E5.4*) and *sHSP* (*hsp‐16.11* and *hsp‐16.2*) in wt animals was reduced by ~80% and ~40%, respectively, between days 1 and 2 of adulthood, the induction of these genes in *eat‐2* mutant animals was not significantly reduced on day 2 of adulthood (*p* > 0.17, *n* > 6) or when compared to the levels seen in wt animals (*p* > 0.12, *n* > 5; Figure 1d and Supporting Information Figure S1c). Thus, two different DR regimens led to the maintenance of robust HS response activation past reproduction onset.

### 2.2 DR alleviates the chronic stress associated with toxic metastable proteins

**Figure 2:**
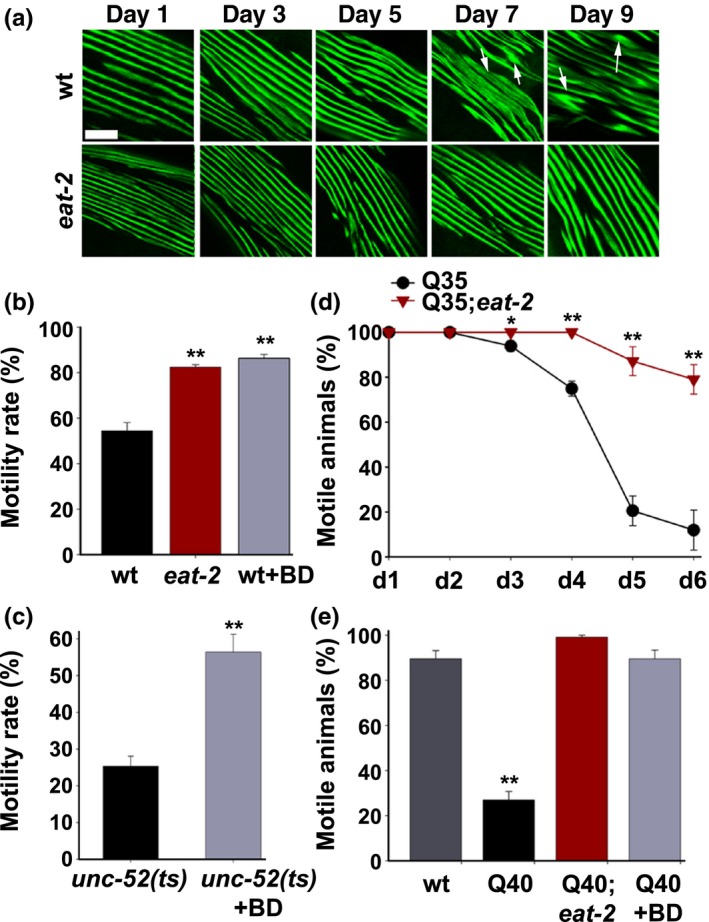
DR modulates folding capacity during adulthood. (a) Confocal images of body‐wall muscle cells. Age‐synchronized wt or *eat‐2(ad453)* animals stained with anti‐MYO‐3 antibodies were fixed and imaged at the indicated times. Arrows point to disrupted myofilaments. Scale bar is 10 µm. (b) Motility of age‐synchronized wt or *eat‐2(ad453)* animals fed ad libitum or BD‐treated for 24 hr. Animal thrashing rates were monitored on day 4 of adulthood. Data were normalized to the motility rate of each strain on day 1 of adulthood. (c) Motility of age‐synchronized *unc‐52(ts)* animals fed ad libitum or BD‐treated for 24 hr. Motility was scored on day 4 of adulthood by determining the percentage of non‐paralyzed *unc‐52(e669,su250) *animals. (d) PloyQ‐associated toxicity of age‐synchronized *Q35m* or *eat‐2(ad453);Q35m* animals. Motility was scored by determining the percentage of non‐paralyzed animals at the indicated times. (e) PloyQ‐associated toxicity of age‐synchronized wt, *Q40n* or *eat‐2(ad453);Q40n* animals fed ad libitum or BD‐treated for 24 hr. Motility was scored by determining the percentage of non‐paralyzed day 6 adult animals. *p *values were calculated by comparison with age‐matched control animals. (**) denotes *p* < 0.01

We next asked whether DR can also enhance the cellular response to chronic expression of misfolded proteins during adulthood. For this, we examined the impact of the *eat‐2* mutation and short BD treatment on the onset of age‐dependent misfolding of metastable or aggregation‐prone proteins expressed in different tissues. Age‐dependent disruption of myofilament organization and declined motility is associated with myosin misfolding in wt animals (Ben‐Zvi et al., 2009; David et al., 2010). The myofilaments of wt animals, examined by monitoring the subcellular localization of myosin heavy chain A (MYO‐3) using specific antibodies were strongly disrupted by day 7 of adulthood. Concomitantly, wt animal motility, measured as thrashing rates, substantially declined by day 4 of adulthood (56 ± 4% of day 1 adults). In contrast, no MYO‐3 disruption was observed in *eat‐*2 mutant animals at least until day 9 of adulthood, with these animals remaining highly mobile (82 ± 1% of day 1 adults; Figure 2a and b). Wild‐type animals also showed high motility rates on day 4 of adulthood (86 ± 2%) following a 24 hr BD treatment (Figure 2b). We next examined the impact of DR on *unc‐52(ts*), the well‐characterized temperature‐sensitive missense mutation in *unc‐52(e669,su250) *that disrupts myofilament anchoring only in adulthood, resulting in age‐dependent stiff body paralysis. While only 25 ± 3% of the *unc‐52(ts)* mutant animals were motile on day 4 of adulthood, the motility of *unc‐52(ts)* animals treated with 24 hr BD treatment was significantly improved (56 ± 5%; Figure 2c). Because of the proximity of *unc‐52(ts) *and *eat‐*2 on the chromosome, we could not obtain double homogenous animals to examine the effects of *eat‐*2 on *unc‐52(ts)*.

Finally, we examined the impact of DR on the toxicity of aggregation‐prone proteins in muscles and neurons. The percent of motile animals expressing glutamine repeats fused to a fluorescent protein in muscle (Q35m) or neurons (Q40n) on the background of wt or *eat‐*2 animals was scored. The onset of Q35m‐mediated paralysis was delayed in Q35;*eat‐2 *animals. By day 6 of adulthood, Q35 animal motility was strongly reduced (12 ± 9%), while 79 ± 7% the Q35;*eat‐2 *animals remained motile (Figure 2d). Q40n‐dependent decline in motility (Shemesh et al., 2017) was also abrogated by DR. By day 6 of adulthood, Q40n animals showed severely reduced motility (27% ± 4%). In contrast, Q40n*;eat‐2* animals, as well as 24 hr BD‐treated Q40n animals, remained completely motile (99 ± 1% and 90 ± 4%, respectively), similar to wt animals (90 ± 4%; Figure 2e). Thus, two different DR regimens resulted in enhanced muscle and neuronal folding maintenance until late in adulthood.

### 2.3 The gonadal longevity pathway and DR differ in their requirements for DAF‐16 and PQM‐1

**Figure 3:**
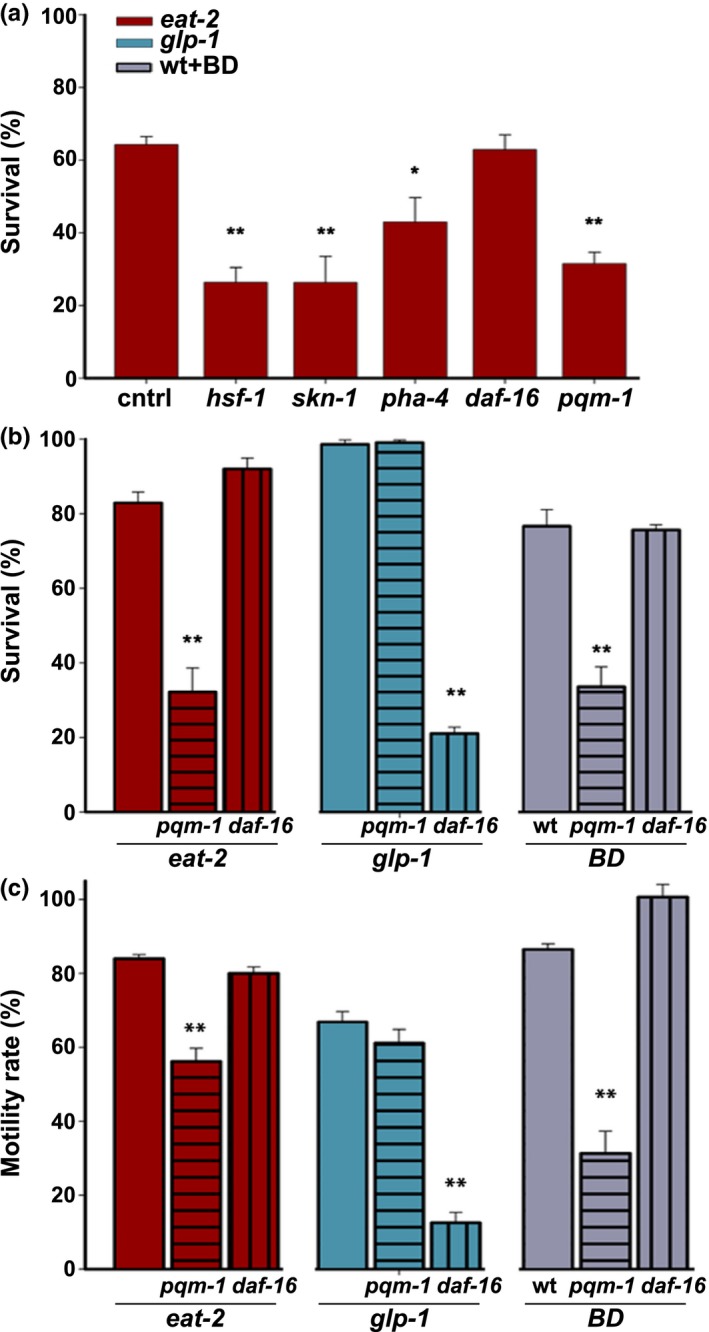
Gonadal signaling and DR diverge on the regulation of DAF‐16 and PQM‐1. (a) Survival rates of age‐synchronized *eat‐2(ad453)* animals fed on the indicated RNAi‐expressing bacteria. Day 3 adult animals were subjected to HS (6 hr at 37°C), and survival was assayed. (b) Survival rates of age‐synchronized *pqm‐1(ok485)* or *daf‐16(mu86)* mutant animals in a wt, *eat‐2(ad453)*, or *glp‐1(e2144) *background*. *Animals were fed ad libitum or BD‐treated for 24 hr and then subjected to HS (6 hr at 37°C); survival was assayed on day 2 of adulthood. (c) Motility of age‐synchronized *pqm‐1(ok485)* or *daf‐16(mu86)* mutant animals in a wt, *eat‐2(ad453)*, or *glp‐1(e2141) *background*. *Animals were fed ad libitum or BD‐treated for 24 hr, and thrashing rates were monitored on day 4 of adulthood. Data were normalized to the strain motility rate on day 1 of adulthood. *p *values were calculated by comparison with age‐matched control animals. (*) denotes *p* < 0.05, and (**) denotes *p* < 0.01

We next examined whether HS activation in *eat‐2* mutant animals is affected by the stress transcription factors, HSF‐1, DAF‐16, PHA‐4, and SKN‐1, similar to GSC‐arrested animals (Berman & Kenyon, 2006; Labbadia & Morimoto, 2015; Lapierre et al., 2011; Shemesh et al., 2013; Steinbaugh et al., 2015). Accordingly, we examined the effect of knocking down these transcription factors using RNA interference (RNAi) on HS survival. While the survival rates of *eat‐2* mutant animals treated with *hsf‐1*, *skn‐1*, or *pha‐4* RNAi were reduced when challenged by HS on day 3 of adulthood, the survival rates of *eat‐2* mutant animals treated with *daf‐16 *RNAi remained high, similar to those of animals challenged with the empty vector control (Figure 3a). This suggests that DR and gonadal signaling differ at least in terms of DAF‐16 requirement. In support, we confirmed previous observations (Tepper et al., 2013) showing that DAF‐16, monitored using a DAF‐16::GFP‐tagged protein construct, is localized to the cytoplasm in *eat‐2* mutants or BD‐treated animals and that genes regulated by DAF‐16 are not activated in *eat‐2* mutant animals (Supporting Information Figure S2). Although gonadal signaling and DR were both affected by HSF‐1, SKN‐1, and PHA‐4 to modulate stress survival, their impacts on these transcription factors could also differ. Indeed, JMJD‐3.1, a methyltransferase upregulated and required for HSF‐1‐dependent HS activation in GSC‐arrested animals (Labbadia & Morimoto, 2015), was not required for BD‐treated *jmjd‐3.1* mutant animals to restore high HS survival rates (Supporting Information Figure S3). Thus, although both DR and gonadal signaling restored stress response activation, there is only partial overlap in the transcription factors that affected HS survival.

Longevity of *eat‐2* mutant animals is also independent of *daf‐16* but was shown to require *pqm‐1* (Dowen, Breen, Tullius, Conery, & Ruvkun, 2016; Lakowski & Hekimi, 1998; Tepper et al., 2013). Nuclear localization and activation of DAF‐16 and PQM‐1 are controlled by IIS and HS in opposite manners. Specifically, when DAF‐16 enters the nucleus, PQM‐1 leaves. We, therefore, asked whether acute and chronic stress responses in DR‐treated animals are dependent on PQM‐1. To answer this question, we crossed *eat‐2* animals with *pqm‐1(ok485)* (*pqm‐1*) or *daf‐16(mu86)* (*daf‐16*) mutant animals and examined the impact on HS survival and motility in adulthood. While the HS survival rates of *eat‐2;daf‐16* animals were unaffected (92 ± 1%), the survival rates of *eat‐2;pqm‐1* mutant animals were strongly reduced (32 ± 6%), similar to those of *pqm‐1 *RNAi‐treated *eat‐2* animals (Figure 3a and b). Likewise, BD treatment enhanced the survival rates of *daf‐16* but not of *pqm‐1 *mutant animals (76 ± 1% and 34 ± 5%, respectively; Figure 3b). In contrast, GSC‐arrested animals required *daf‐16*, as previously observed (Shemesh et al., 2013), but not *pqm‐1*. The survival of *glp‐1(e2144) *animals *(glp‐1) *crossed with *daf‐16 *animals was essentially abolished (21 ± 2%), whereas the survival rates of *glp‐1 *animals crossed with *pqm‐1* animals were unaffected (99 ± 1%; Figure 3b). A similar pattern of *pqm‐1 *and *daf‐16 *dependence was observed for animal motility. The motility of *eat‐2;daf‐16* mutant animals on day 4 of adulthood was similar to that of *eat‐2* mutant animals (80 ± 2% and 84 ± 1%, respectively), while the motility of BD‐treated *daf‐16* mutant animals was similar to that of BD‐treated wt animals (100 ± 2% and 86 ± 2%, respectively; Figure 3c). In contrast, the motility of *eat‐2;pqm‐1* mutant animals and BD‐treated *pqm‐1* mutant animals was strongly reduced (56 ± 4% and 31 ± 6%, respectively). Complementing these results, the motility of *glp‐1;daf‐16* animals was strongly reduced (13 ± 3%), while the motility of *glp‐1*;*pqm‐1* animals was similar to that of *glp‐1* animals (61 ± 4% and 67 ± 3%, respectively; Figure 3c). Thus, the gonadal longevity pathway and DR modulated quality control functions through the antagonistic transcription factors, DAF‐16 and PQM‐1, respectively.

### 2.4 PQM‐1 and DAF‐16 can establish different chaperone networks

**Figure 4:**
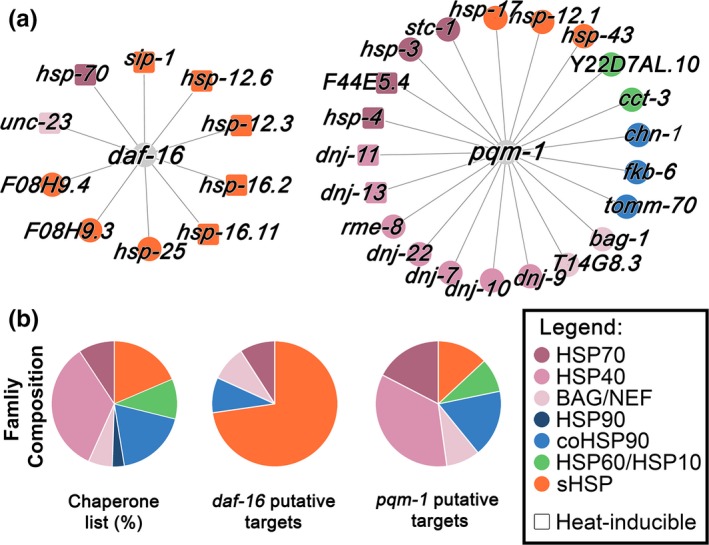
Putative chaperone targets of DAF‐16 and PQM‐1 can differentially modulate the chaperone network. (a) Putative DAF‐16 and PQM‐1 chaperone targets. Chaperone families are indicted by color. Heat‐inducible chaperones are marked by a square. (b) Distribution of chaperone genes by chaperone families for the chaperone list (97 genes), the putative *daf‐16* targets list (10 genes) or the putative *pqm‐1* targets list (21 genes)

Meta‐analysis of DAF‐16 expression profiles using DAF‐16 target ranking suggests that DAF‐16 regulates the expression of DAF‐16‐binding element (DBE) genes, inducing a cellular stress response. PQM‐1, in turn, is required for the transcription of a DAF‐16‐associated element (DAE) linked to growth and development (Tepper et al., 2013). Given that one of the main differences between gonadal signaling and DR regulation was DAF‐16 or PQM‐1 dependency, we next asked how these transcription factors could affect the chaperone network. For this, we sought chaperones in the DAF‐16 and PQM‐1 putative target lists found in Tepper et al. (2013). Putative PQM‐1 targets include a representative distribution of the different chaperone families (21 of 97 chaperones; Figure 4a and b), although *hsp‐90*, a known PQM‐1 target (O'Brien et al., 2018), did not pass the set false discovery rate (FDR) cutoff. PQM‐1‐associated chaperones are expressed in different cellular compartments and are linked to de novo folding, protein transport, and endocytosis, supporting a general role in folding maintenance (Supporting Information Table S1). In contrast, putative DAF‐16 targets include 10 cytosolic chaperones that are highly enriched in sHSP (Supporting Information Table S1), encompassing 8 of the 18 sHSP genes in *C. elegans* (*p* > 0.00001; Figure 4a and b). To examine the predicted distribution of putative PQM‐1 and DAF‐16 targets between cognate and stress‐inducible chaperones, we assembled a list of heat‐induced chaperones (22 of 97; Supporting Information Table S2) based on two independent RNA‐seq experiments (Brunquell, Morris, Lu, Cheng, & Westerheide, 2016; Li, Chauve, Phelps, Brielmann, & Morimoto, 2016). This list is enriched in sHSP genes (9 of 22, *p* > 0.004), as well as Hsp70 genes (5 of 9, *p* > 0.022; Supporting Information Table S2). Heat‐inducible chaperones are typically represented in the list of putative PQM‐1 targets (4 of 23 genes). In contrast, most of the putative DAF‐16 target chaperones are heat‐inducible (7 of 10; *p* < 0.001; Supporting Information Table S1). We previously demonstrated that expression levels of *hsp‐16.2 *and *hsp‐70 *but not of *F44E5.4 *are significantly reduced in *glp‐1 *animals treated with *daf‐16 *RNAi (Shemesh et al., 2013). To validate putative PQM‐1 targets, we compared chaperones basal and induced levels in *eat‐2 *and *eat‐2;pqm‐1 *mutants animals, using qPCR. *F44E5.4 *and *hsp‐16.11 *induction (10^2^–10^3^ fold increase) was unaffected in *eat‐2;pqm‐1 *mutant animals, in agreement with PQM‐1 translocation out of the nucleus upon HS (Tepper et al., 2013). In contrast, basal levels of *F44E5.4* but not of *hsp‐16.11* were increased 10‐fold in *eat‐2;pqm‐1* mutant animals, as compared to *eat‐2* animals, suggesting that *F44E5.4* is negatively regulated by PQM‐1 in an *eat‐2* background (Supporting Information Figure S4a and b). Moreover, basal expression levels of *fkb‐6 *but not of *hsp‐25 *were decreased by ~30% in *eat‐2;pqm‐1* mutant animals, as compared to *eat‐2* animals, suggesting that *fkb‐6 *is positively regulated by PQM‐1 in the *eat‐2* background (Supporting Information Figure S4b). Thus, while both gonadal signaling and DR enhance quality control functions, our analysis suggests that DAF‐16‐dependent chaperone regulation is likely geared toward enhanced survival and recovery from acute stresses, whereas PQM‐1‐dependent chaperone regulation is likely to support maintenance and therefore withstanding the chronic expression of misfolded proteins.

### 2.5 Differential modulation of the quality control network capabilities

**Figure 5:**
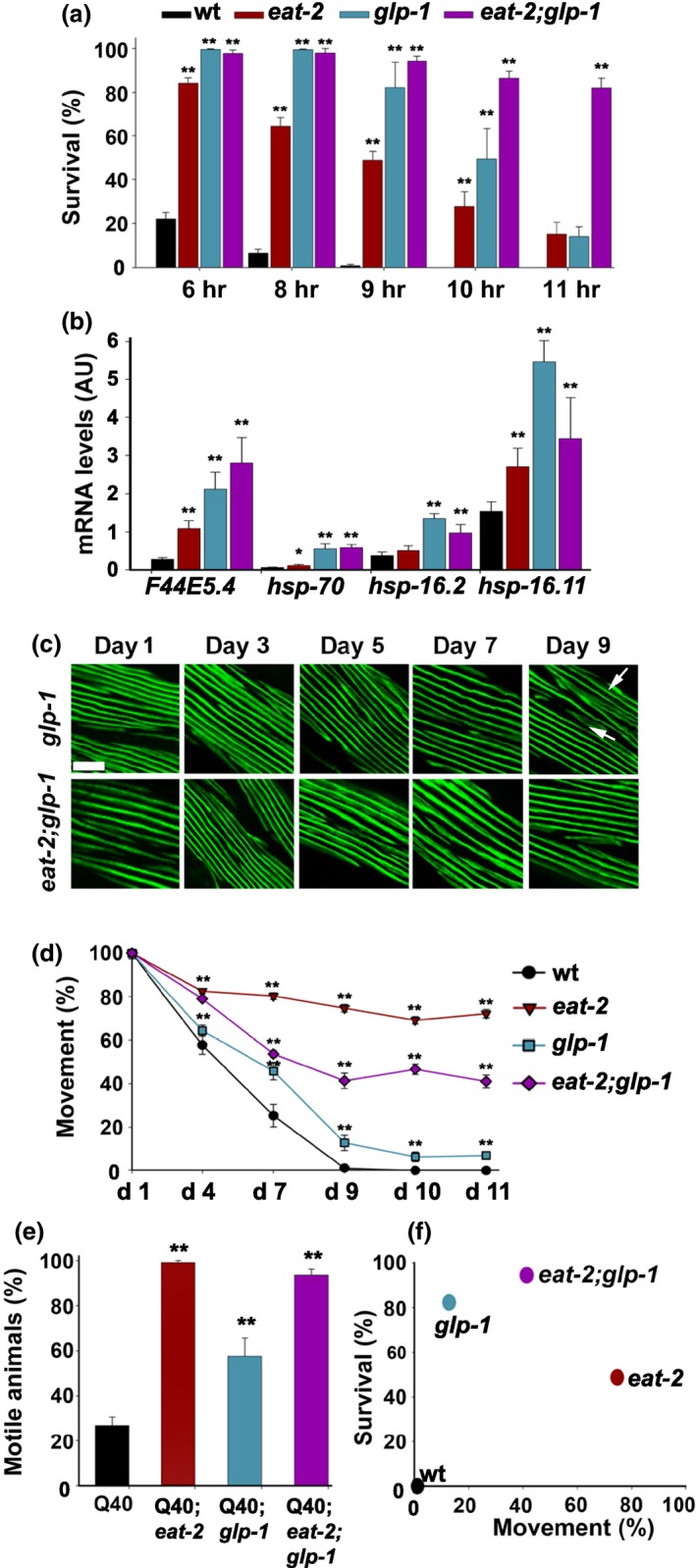
Comparing acute and chronic stress capabilities of *eat‐2* and *glp‐1* mutant animals. (a) Survival rates of age‐synchronized wt, *eat‐2(ad453)*, *glp‐1(e2144), *or* eat‐2(ad453)*;*glp‐1(e2144) *animals. Day 2 adult animals were subjected to HS (6–11 hr at 37°C), and survival was assayed. (b) Expression levels of HS genes. mRNA levels of HS genes, as indicated, from age‐synchronized wt, *eat‐2(ad453)*, *glp‐1(e2144), *or *eat‐2(ad453)*;*glp‐1(e2144) *animals subjected to HS (90 min at 37°C). (c) Confocal images of body‐wall muscle cells. Age‐synchronized *glp‐1(e2144) *or* eat‐2(ad453)*;*glp‐1(e2144)* animals stained with anti‐MYO‐3 antibodies were fixed and imaged at the indicated times. Arrows point to disrupted myofilaments. Scale bar is 10 µm. (d) Motility of age‐synchronized wt, *eat‐2(ad453)*, *glp‐1(e2144)*, or* eat‐2(ad453)*;*glp‐1(e2144) *animals. Animal thrashing rates were monitored on days 1–11 of adulthood. Data were normalized to the motility rate of each strain on day 1 of adulthood. (e) PolyQ‐associated toxicity of age‐synchronized *Q40n*, *eat‐2(ad453);Q40n glp‐1(e2144);Q40n*, or *eat‐2(ad453)*;*glp‐1(e2144);Q40n *mutant animals. Motility was scored by determining the percentage of non‐paralyzed day 6 adult animals. (f) Survival rates (9 hr) plotted against motility rates (day 9). *p *values were calculated by comparison with age‐matched control animals. (*) denotes *p* < 0.05, and (**) denotes *p* < 0.01

To test our prediction that GSC‐dependent regulation is more likely to enhance stress activation than is DR, we first compared *eat‐2* and *glp‐1* mutant animals under extended HS conditions. While survival rates of *eat‐2* animals declined by ~25% following 6‐8 hr of stress, survival rates of *glp‐1* animals only showed a similar decline after 10 hr of HS (Figure 5a), demonstrating the more robust HS response of GSC‐arrested animals. In agreement, the expression levels of tested HS genes examined by qPCR were two‐ to fourfold higher in *glp‐1* than in *eat‐2 *mutant animals after HS on day 2 of adulthood (Figure 5b). In contrast, when we compared the impacts of the *eat‐2* and *glp‐1* mutations on the age‐dependent decline in motility, we found that the thrashing rates of *glp‐1* mutant animals completely declined by day 9 of adulthood (13 ± 4% of day 1), whereas *eat‐2* animals maintained high motility rates even at day 10 of adulthood (69 ± 2% of day 1). Likewise, myosin organization was mildly affected in *glp‐1* but not in *eat‐2* mutant animals by day 9 of adulthood (Figure 5 c and d). This suggests that *eat‐2* animals have a more robust maintenance capacity than do *glp‐1* animals. In agreement, the motility of Q40n*;eat‐2* animals was higher on day 6 of adulthood than that of Q40n*;glp‐1* animals (99 ± 1% and 58 ± 8%, respectively; Figure 5e). Thus, DR and gonadal signaling both remodel quality control networks in adulthood, although their capacity and function differ (Figure 5f).

### 2.6 DR further improves quality control functions of GSC‐arrested animals in a daf‐16‐dependent manner

Given that DR did not further improve GSC‐dependent lifespan extension (Crawford, Libina, & Kenyon, 2007) and that DAF‐16 and PQM‐1 are antagonistic (Tepper et al., 2013), we next asked whether and how activating both pathways would impact quality control functions. For this, we crossed *eat‐2* and *glp‐1* mutant animals (*eat‐2;glp‐*1) and examined their responses to acute or chronic folding stresses. The response of *eat‐2;glp‐*1 animals to folding challenges resembled that of *glp‐1* animals. The survival rates of *eat‐2;glp‐1* mutant animals were high and maintained during adulthood (Supporting Information Figure S5a). The survival rates of *eat‐2;glp‐*1 animals were high even after a prolonged HS (10 hr, 37°C; 87 ± 3%) and could be associated with a strong induction of the tested HS genes (Figure 5a and b). Likewise, the thrashing rates of *eat‐2;glp‐*1 animals declined with age, although the decline was milder and myosin organization was unaffected until day 9 of adulthood (Figure 5 c and d). These results suggest that gonadal signaling is the main driver of proteostasis remodeling in these animals. In agreement, DAF‐16::GFP showed strong nuclear localization in the background of *eat‐2;glp‐1* mutant animals and induced the expression of DAF‐16‐dependent targets, such as *sod‐3* and *hsp‐16.2* (Supporting Information Figure S2). Moreover, the survival rate of *eat‐2;glp‐1 *animals harboring a *daf‐16* mutation was reduced, whereas their motility rate was improved (Supporting Information Figure S5b and c). However, the motility of *eat‐2;glp‐1 *animals, as well as that of Q40;*eat‐2;glp‐1 *animals, was significantly improved, as compared to that of *glp‐1* animals (Figure 5d and e). This suggests that additional factors are required for proteostasis remodeling in *eat‐2;glp‐1* animals. Moreover, the survival rates of *eat‐2;glp‐1 *treated with *hsf‐*1, *skn‐1* or *pha‐4* RNAi were reduced (Supporting Information Figure S5d), suggesting a role for these factors in both pathways. These data indicate that the quality control network of *eat‐2;glp‐*1 mutant animals is determined mainly by gonadal signaling and requires DAF‐16. However, DR can further improve both the acute and chronic responses of GSC‐arrested animals (Figure 5f).

## 3 DISCUSSION

Aging is associated with the accumulation of damaged and misfolded proteins through a functional decline in cellular quality control machineries (Riera et al., 2016; Sala et al., 2017; Shai et al., 2014). Signaling pathways that modulate aging progression, such as the IIS, gonadal signaling, or DR, can regulate quality control networks to restore seemingly youthful proteostasis. Here, we addressed the rapid decline in acute and chronic stress responses at the transition to reproductive adulthood (Labbadia & Morimoto, 2015; Shemesh et al., 2013) to ask whether DR can restore quality control in adulthood and to compare the impact of two different signaling pathways on quality control functions.

**Figure 6:**
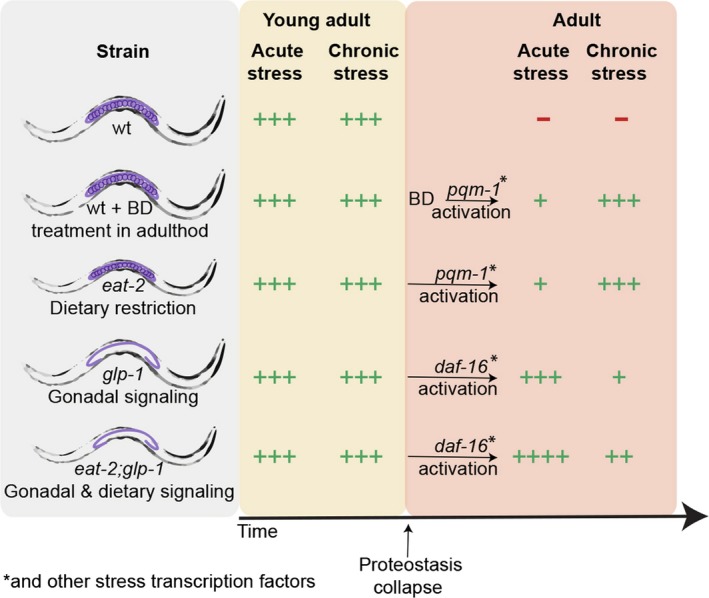
Quality control networks are differentially remodeled by gonadal and dietary signals. In wt animals, proteostasis collapses at the transition to adulthood. Gonadal and dietary signals differentially remodel the quality control network; gonadal signaling enhances acute stress responses, while DR enhances the response to chronic stresses

We first demonstrated, using two different DR regimens (McKay et al., 2004; Steinkraus et al., 2008), that dietary signaling can remodel quality control in adulthood, rescuing stress activation, and protein folding capacity. However, it is also possible that *eat*‐2 mutation that affects the function of an acetylcholine receptor modulates quality control networks cell non‐autonomously. Directly comparing acute and chronic stress responses in DR and GSC‐arrested animals pointed to at least two different strategies of remodeling quality control networks. Whereas DR mildly improved HS survival rates, it transformed folding maintenance and the response to chronic misfolding stress. In contrast, signaling from the gonad rescued HS response activation while only mildly modulating folding maintenance and the response to chronic misfolding. We thus propose that different remodeling pathways reshape the quality control network, rather than restore it to the youthful state (Figure 6). Such network remodeling will have implications on cellular ability to withstand acute or chronic stresses and thus could impact vulnerability to diseases associated with the age‐dependent accumulation of damage proteins.

### 3.1 Shared and unique aspects of quality control regulation

Our observations indicate that DR and the gonadal longevity pathway enhanced quality control networks independently, at least in part. Indeed, the commitment to network remodeling by gonadal signaling occurs during larval development (Shemesh et al., 2017). At the same time, DR‐dependent network remodeling was induced by BD treatment post‐development and even late in adulthood. It will be interesting to examine until when and to what extent BD can modulate folding maintenance, as exemplified by myosin organization or polyQ‐dependent toxicity. Second, DR‐dependent rescue required PQM‐1, rather than DAF‐16. We propose that the downstream targets of these two transcription factors can account, in part, for the differences in the folding capacities seen in adulthood (Figure 6). For example, the putative chaperone targets of DAF‐16 are sHSP that can bind misfolded proteins and associate into large complexes to modulate aggregate features rather than to induce their removal (Bar‐Lavan et al., 2016). In agreement, Cohen, Bieschke, Perciavalle, Kelly, and Dillin (2006) noted that HSF‐1 and DAF‐16 regulation could impact cellular response to aggregation, with HSF‐1 supporting disaggregation and DAF‐16‐inducing aggregation. Moreover, PQM‐1 was recently found to be linked to transcellular stress response activation that mediates chaperone regulation non‐autonomously in response to the accumulation of damage proteins in different tissues (O'Brien et al., 2018). A wider and more direct analysis of DAF‐16 and PQM‐1 targets is, therefore, required. Third, although DR‐mediated effects on the quality control network were affected by several stress transcription factors that are also recruited by the gonadal longevity pathway, including HSF‐1, PHA‐4, and SKN‐1, their impact on the network could differ. Indeed, while both IIS and gonadal signaling activate DAF‐16, their impacts on DAF‐16 localization or activation, DAF‐16 cofactors and target selection differ (Antebi, 2013; Riera et al., 2016). For example, GSC‐arrested animals require KRI‐1 for DAF‐16 activation, while IIS does not (Berman & Kenyon, 2006). In agreement, we showed that DR, similar to gonadal signaling, was unable to restore HS activation in adulthood in *hsf‐1(RNAi)*‐treated animals, even though HS induction levels, as well as the requirement for *jmjd‐3.1*, differed between the two processes. Finally, the difference in quality control capabilities could also be due to other regulatory elements or pathways not explored here, such as nuclear hormone receptor DAF‐12 that functions in the gonadal pathway (Antebi, 2013; Shemesh et al., 2013). Thus, a combination of non‐autonomous signals can modulate different aspects of damage responses, likely affecting an organism's capacity to cope with acute and chronic stresses.

One interesting observation from our work is that the choice between DAF‐16 and PQM‐1 activation was not determined by diet, as the requirement for DAF‐16 differed between intact and GSC‐arrested animals. This suggests that signals from the reproductive system determine DAF‐16 or PQM‐1 localization and activation under ad labium and DR conditions that can have implication on fat metabolism and transport (Dowen et al., 2016). Crosstalk between reproduction and somatic maintenance is likely to respond to environmental conditions that impact reproductive success, such as food availability (Hubbard, Korta, & Dalfo, 2013). This suggests that differences in DAF‐16 or PQM‐1 requirement can be accounted for by the distinct impacts of different DR protocols on the reproductive system. Indeed, steroid signaling mediated by the nuclear hormone receptor NHR‐8 in response to nutrient scarcity affects germline plasticity and longevity (Thondamal, Witting, Schmitt‐Kopplin, & Aguilaniu, 2014). It will be interesting to examine how DR regimens that were shown to require *daf‐16* impact the germline and quality control functions.

### 3.2 Cell non‐autonomous regulation of acute and chronic stresses

We found that activation of the gonadal longevity pathway contributes to robust stress response activation and survival, while DR prolonged folding maintenance and the capacity to withstand chronic stresses. This observation strengthens the emerging notion that the ability to respond to acute stress could come at the expense of the response to chronic stress (Maman et al., 2013; Prahlad & Morimoto, 2011). Neuronal regulation of the HS response was shown to inversely regulate acute and chronic responses to damage protein accumulation. While disrupting the ability of thermosensory neurons to detect temperature change inhibited HS gene induction under acute stress conditions, it enhanced an animal's ability to cope with chronic expression of metastable or aggregation‐prone proteins (Prahlad & Morimoto, 2011). Moreover, the activation of different transcription factors, such as DAF‐16, HSF‐1, PHA‐4, and PQM‐1, was shown to be differentially regulated as a function of the source of the accumulating damage (Maman et al., 2013; O'Brien et al., 2018). It will be most interesting to connect our observations on dietary and gonadal signaling impact on acute and chronic stresses with specific neuronal regulation pathways.

Finally, it remains unclear how the regulation of acute and chronic stress activation is linked to lifespan. No further improvement to lifespan was observed between intact and GSC‐arrested *eat‐2 *animals (Crawford et al., 2007), although both acute and chronic responses were enhanced in *eat‐2;glp‐1* animals (Figure 5f). A similar dissociation between healthspan and lifespan was observed in other pathways and systems (Dubnikov, Ben‐Gedalya, & Cohen, 2017), suggesting that healthspan regulation contributes but is not the only determent to enhanced lifespan. One possibility is that any impact on lifespan would depend both on the capacity of the system and the hormetic effects of stress on the organism's life history.

## 4 EXPERIMENTAL PROCEDURES

### 4.1 Nematode strains and growth conditions

A list of strains used in this work is provided in Supporting Information Table S3. Mutant strains were outcrossed into our N2 strain (*n* > 3). We used standard genetic crossing techniques to construct and verify double or triple mutants. *C. elegans* was cultured using standard techniques; nematodes were grown on NGM plates seeded with the *Escherichia coli* OP50‐1 strain at 15°C and fed ad libitum. For BD, age‐synchronized adults were transferred to NGM agar plates without a lawn of *E. coli *24 hr prior to being scored. Unless otherwise stated, ~40 embryos, laid at 15°C, were transferred to fresh plates and grown at 25°C for the duration of the experiment. The first day of adulthood was set at 50 hr after temperature shift, before the onset of egg laying. Animals were moved during the reproductive period to avoid progeny contamination.

### 4.2 Bioinformatics and statistics

The chaperone list was previously compiled based on the work of Brehme et al. (2014), focusing on the main chaperone and cochaperones families (97 genes) (Bar‐Lavan, Shemesh, Dror, et al., 2016). For the putative chaperone target lists (Supporting Information Table S1), the curated list of putative DAF‐16 and PQM‐1 targets at a 5% FDR (1,663 positive DAF‐16‐associated targets and 1,733 negative PQM‐1‐associated targets) provided in Tepper et al. (2013) as Supporting Information was used. For the HS‐induced chaperone list (Supporting Information Table S2), two curated lists of RNA‐seq transcripts significantly induced by HS were used: (a) wt (N2) L2 larvae grown at 20°C treated or untreated by HS (34°C for 30 min), kindly provided by Dr. Jian Li and Dr. Richard Morimoto (Li et al., 2016); (b) wt (N2) L4 larvae grown at 23°C treated or untreated by HS (33°C for 30 min), provided by Brunquell et al. (2016) as Supporting Information. The probability of overlap between the chaperone sets was calculated using the Fisher exact test. Experiments were repeated at least three times, and >30 animals were scored per experimental condition. Data are presented as means ± SEM. *p* values were calculated using the Mann–Whitney rank sum test to compare two independent populations, where (*) denotes *p* < 0.05 and (**) denotes *p* < 0.01.

### 4.3 HS assays

For each HS treatment assay, a total of ~30 age‐synchronized animals grown at 25°C were used. Animals were moved to fresh plates and placed in a 37°C bath for 90 min. Animals were frozen or fixed immediately following the HS.

To determine HS survival rates, age‐synchronized animals were transferred to a 24‐well plate containing HS buffer (100 mM Tris–HCl, pH 7.4, 17 mM NaCl and 1% cholesterol supplemented with bacteria). Animals were subjected to a 37°C HS for 6 hr, unless otherwise indicated. HS buffer was supplemented with SYTOX orange (Invitrogen, Carlsbad, CA), and animal survival (*n* > 85) was scored by monitoring dye uptake using a Leica M165 FC fluorescent stereoscope with a TXR filter. Fluorescent animals were scored as dead.

Stress reporters were examined by employing GFP_HS_‐expressing animals. Animals were subjected to HS, and fluorescence was monitored 18–24 hr later. Animals expressing GFP in the gut were scored as HS‐induced. >65 animals per experimental condition were scored.

### 4.4 Feeding RNA interference

Embryos were placed on *E. coli* strain HT115(DE3) transformed with the indicated RNAi vectors (obtained from the Ahringer or Vidal RNAi libraries), as previously described (Shemesh et al., 2013). RNAi knockdown efficiency varied between experiments (~40%–80%).

### 4.5 Determination of RNA levels

RNA extraction from synchronized animals, cDNA synthesis, and quantitative real‐time PCR was performed as previously described (Shemesh et al., 2017). Samples were normalized (using the 2‐ΔΔCT method) to *act‐1* or 18S levels. The list of primers used in this work is provided in Supporting Information Table S4.

### 4.6 Immunostaining

Adult animals were fixed with 4% paraformaldehyde and permeabilized with β‐mercaptoethanol and collagenase IV treatment, as described (Bar‐Lavan, Shemesh, Dror, et al., 2016). Anti‐MYO‐3 (5–6) and secondary DyLight 488 anti‐mouse antibodies (Jackson Immuno Research) were used. Adult animals expressing fluorescence reporters were fixed with 4% paraformaldehyde. Treated samples were imaged using a Leica DM5500 confocal microscope with 488 nm laser lines for excitation or with a Leica DFC360FX camera.

### 4.7 Motility assays

To determine thrashing rates, animals (*n* > 30) were placed in wells containing M9 buffer. After a 5 min acclimation period, each animal was monitored for 15 s and thrashes (i.e., changes in the direction of bending at mid‐body) were counted. Values are presented as bends per minute.

To determine the percent of motile animals, animals (*n* > 30) grown at 25°C, were moved every day, and paralysis was scored by monitoring animal movement 10 min after transfer to a new plate. Animals that did not move were scored as paralyzed.

To determine *unc‐52(ts)* motility, day 1 adult *unc‐52(ts)* mutant animals, grown at 25°C, were shifted to 15°C and motility was scored on day 4 of adulthood as above.

## AUTHOR CONTRIBUTIONS

ABZ and N. Shpigel designed the study. N. Shpigel and MK performed the experiments, and N. Shemesh performed the bioinformatic analyses. ABZ, N. Shpigel, and N. Shemesh wrote the manuscript. ABZ acquired funding and supervised the experiments.
