# Supplementary figures and images for: Dietary restriction and gonadal signaling differentially regulate post‐development quality control functions in Caenorhabditis elegans
Source: Aging Cell. 2019 Jan 15;18(2):e12891. doi: 10.1111/acel.12891 (PMC6413660; doi:10.1111/acel.12891)

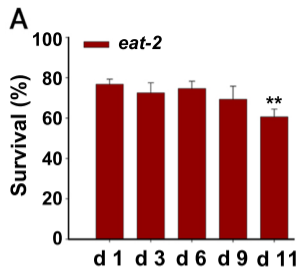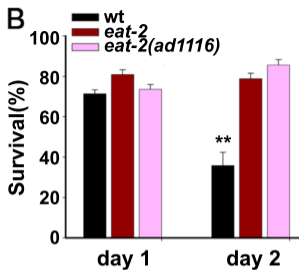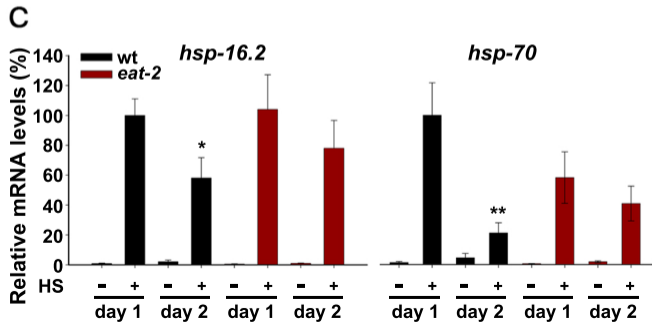

Supplement: Supplementary file 1 [file ACEL-18-e12891-s001.pdf]

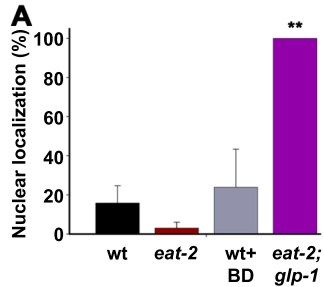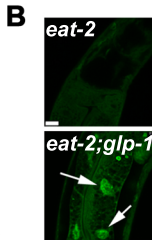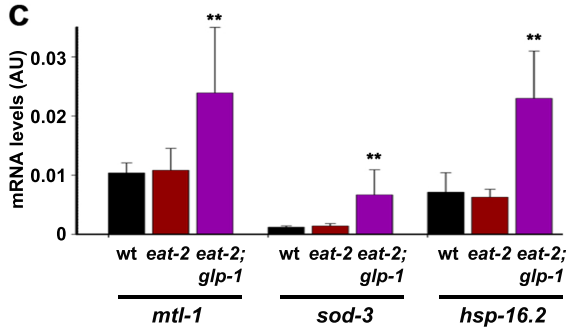

Supplement: Supplementary file 2 [file ACEL-18-e12891-s002.pdf]

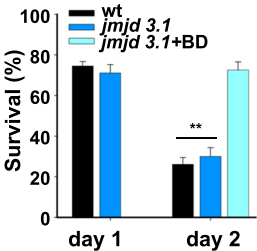

Supplement: Supplementary file 3 [file ACEL-18-e12891-s003.pdf]

**A**

Relative mRNA levels (%)

HS

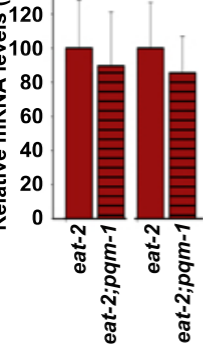

F44E5.4

*hsp16.11***B**  
Relative mRNA levels (%)

non HS

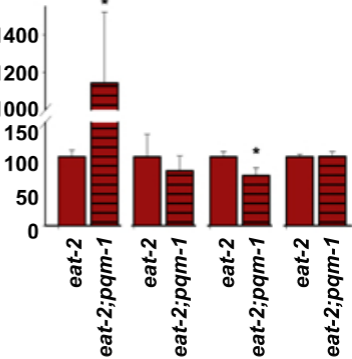

F44E5.4

*hsp16.11**fkb-6**hsp-25*

Supplement: Supplementary file 4 [file ACEL-18-e12891-s004.pdf]

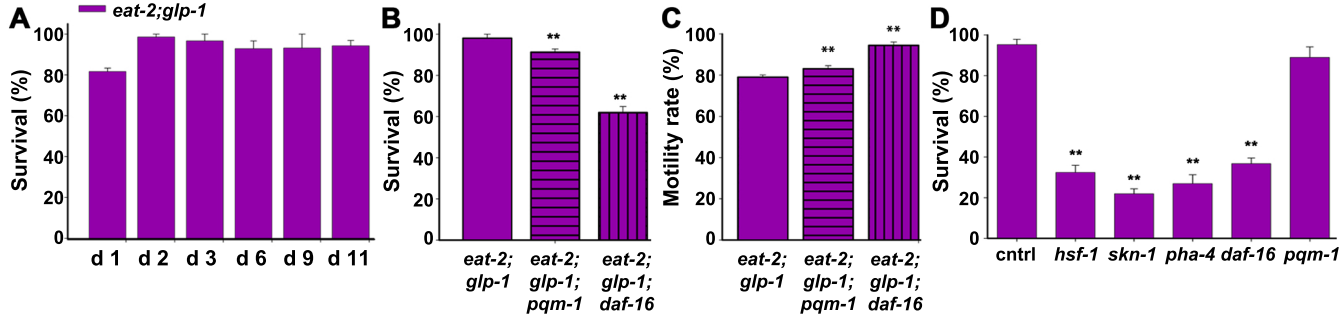

Supplement: Supplementary file 5 [file ACEL-18-e12891-s005.pdf]
