# Supplementary material for: Dietary restriction and gonadal signaling differentially regulate post‐development quality control functions in Caenorhabditis elegans
Source: Aging Cell. 2019 Jan 15;18(2):e12891. doi: 10.1111/acel.12891 (PMC6413660; doi:10.1111/acel.12891)
Supplement: Supplementary file 6 [file ACEL-18-e12891-s006.pdf]

Table S1. Putative gene targets of DAF-16 and PQM-1

Genes	Chaperone family	Compartment	Heat-induced
**DAF-16 Putative targets**			
hsp-70	HSP70	Cytosol	+
unc-23	NEF	Cytosol	+
hsp-16.2	sHSP	Cytosol	+
hsp-12.6	sHSP	Cytosol	+
sip-1	sHSP	Cytosol	+
hsp-12.3	sHSP	Cytosol	+
hsp-16.11	sHSP	Cytosol	+
hsp-25	sHSP	Cytosol	+
F08H9.3	sHSP	Cytosol	
F08H9.4	sHSP	Cytosol	
**PQM-1 putative targets**			
F44E5.4	HSP70	Cytosol	+
hsp-3	HSP70	ER	
hsp-4	HSP70	ER	+
stc-1	HSP70	ER	
dnj-7	HSP40	ER	
dnj-9	HSP40	Cytosol	
dnj-10	HSP40	Mitochondria	
dnj-11	HSP40	Cytosol / Ribosomes	+
dnj-13	HSP40	Cytosol	+
dnj-22	HSP40	Cytosol	
rme-8	HSP40	Cytosol / Endosomes	
bag-1	NEF	Cytosol	
T14G8.3	NEF	ER	
fkb-6	coHSP90	Cytosol	
chn-1	coHSP90	Cytosol	
tomm-70	coHSP90	Mitochondria	
Y22D7AL.10	HSP60	Mitochondria	
cct-3	HSP60	Cytosol	
hsp-43	sHSP	Cytosol	
hsp-17	sHSP	Cytosol	
hsp-12.1	sHSP	Cytosol	
