# Supplementary material for: Dietary restriction and gonadal signaling differentially regulate post‐development quality control functions in Caenorhabditis elegans
Source: Aging Cell. 2019 Jan 15;18(2):e12891. doi: 10.1111/acel.12891 (PMC6413660; doi:10.1111/acel.12891)
Supplement: Supplementary file 7 [file ACEL-18-e12891-s007.pdf]

Table S2. Heat-inducible chaperones

Genes	Chaperone family	Compartment	Brunquell *et al.*, 2016	Li *et al.*, 2017
hsp-1	HSP70	Cytoplasm	+	+
hsp-4	HSP70	ER	+	+
hsp-70	HSP70	Cytoplasm	+	+
F44E5.4	HSP70	Cytoplasm	+	+
F44E5.5	HSP70	Cytoplasm	+	+
dnj-11	HSP40	Cytoplasm		+
dnj-13	HSP40	Cytoplasm	+	+
K07F5.16	HSP40	Cytoplasm		+
unc-23	NEF	Cytoplasm	+	+
hsp-110	NEF	Cytoplasm	+	+
daf-21	HSP90	Cytoplasm	+	
C01G10.8	coHSP90	Cytoplasm		+
sti-1	coHSP90	Cytoplasm	+	
hsp-12.3	sHSP	Cytoplasm		+
hsp-12.6	sHSP	Cytoplasm	+	
sip-1	sHSP	Cytoplasm		+
hsp-16.1	sHSP	Cytoplasm	+	+
hsp-16.11	sHSP	Cytoplasm	+	+
hsp-16.2	sHSP	Cytoplasm	+	+
hsp-16.41	sHSP	Cytoplasm	+	+
hsp-16.48	sHSP	Cytoplasm	+	
hsp-16.49	sHSP	Cytoplasm	+	+
