# Supplementary material for: Dietary restriction and gonadal signaling differentially regulate post‐development quality control functions in Caenorhabditis elegans
Source: Aging Cell. 2019 Jan 15;18(2):e12891. doi: 10.1111/acel.12891 (PMC6413660; doi:10.1111/acel.12891)
Supplement: Supplementary file 8 [file ACEL-18-e12891-s008.pdf]

Table S3. List of strains and abbreviations used in this work

Strain	Abbreviation	Genotype
N2	*wild type (wt)*	---
DA453	*eat-2*	*eat-2(ad453) II*
DA1116	*eat-2*	*eat-2(ad1116) II*
CF1903	*glp-1*	*glp-1(e2141ts)*
ABZ95	*eat-2:glp-1*	*eat-2(ad453) II; glp-1(2141ts)*
ABZ22*	GFP_{HS}	*dvIs70[phsp-16.2::gfp; rol-6(su1006)]*
ABZ135	*eat-2;*GFP_{HS}	*eat-2(ad453) II ;dvIs70[phsp-16.2::gfp; rol-6(su1006)]*
ABZ23	*glp-1;*GFP_{HS}	*glp-1(2141ts); dvIs70[phsp-16.2::gfp; rol-6(su1006)]*
ABZ136	*eat-2;glp-1;*GFP_{HS}	*eat-2(ad453) II; glp-1(2141ts);dvIs70[phsp-16.2::gfp; rol-6(su1006)]*
HE250	*unc-52 (ts)*	*unc-52(e669su250)*
ABZ20*	Q35m	*rmIs132[punc-54::q35::yfp]*
ABZ137	Q35m;*eat-2;*	*eat-2(ad453);rmIs132[punc-54::q35::yfp]*
AM47	Q40n	*RmIs167[pF25B3.3::q40::cfp]*
ABZ214	Q40n;*eat-2*	*eat-2(ad453) II; RmIs167[pF25B3.3::q40::cfp]*
ABZ69	Q40n;*glp-1*	*glp-1(2141ts); RmIs167[pF25B3.3::q40::cfp]*
ABZ216	Q40n;*eat-2;glp-1*	*eat-2(ad453) II; glp-1(2141ts); RmIs167[pF25B3.3::q40::cfp]*
CF1038	*daf-16*	*daf-16(mu86)*
ABZ220	*eat-2;daf-16*	*eat-2(ad453) II; daf-16(mu86)*
CF1880	*glp-2;daf-16*	*glp-1(e2141); daf-16(mu86)*
ABZ213*	*pqm-1*	*pqm-1(ok485) II*
ABZ218	*eat-2;pqm-1*	*eat-2(ad453) II; pqm-1(ok485) II*
ABZ219	*glp-2;pqm-1*	*glp-1(2141ts);pqm-1(ok485) II*
ABZ226	*eat-2:glp-1:daf-16*	*eat-2(ad453) II; glp-1(2141ts); daf-16(mu86)*
ABZ221	*eat-2:glp-1:pqm-1*	*eat-2(ad453) II; glp-1(2141ts); pqm-1(ok485) II*
TJ356	*daf-16:GFP*	*daf-16p::daf-16a/b::GFP + rol-6(su1006)*
ABZ223	*eat-2:daf-16:GFP*	*eat-2(ad453) II; daf-16p::daf-16a/b::GFP + rol-6(su1006)*
ABZ225	*eat-2:glp-1:daf16:GFP*	*eat-2(ad453) II; glp-1(2141ts);daf-16p::daf-16a/b::GFP + rol-6(su1006)*
ZR2	*jmjd 3.1*	*jmjd-3.1(gk384)*

*Strains were out-crossed three times to our laboratory N2 stock.
