# Supplementary material for: Dietary restriction and gonadal signaling differentially regulate post‐development quality control functions in Caenorhabditis elegans
Source: Aging Cell. 2019 Jan 15;18(2):e12891. doi: 10.1111/acel.12891 (PMC6413660; doi:10.1111/acel.12891)
Supplement: Supplementary file 9 [file ACEL-18-e12891-s009.pdf]

Table S4. List of primer sequences used in this work

	F'	R'
actin	ATCACCGCTCTTGCCCCATC	GGCCGGACTCGTCGTACTCTTG
hsp16.2	ACTTTACCACTATTTCCGTCCAGC	CCTTGAACCGCTTCTTTCTTTG
hsp16.11	CCACTATTTCCGTCCAGCTC	GCTTGAACTGCGAGACATTG
c12c8.1	CTACATGCAAAGCGATTGGA	TGTAAAAGCCACGTAGGAAGG
F44E5.4	GCAAAGCTATTGGTATCGAC	CACGTATGATGGAGTTGTCTTA
daf-16 mut	TTCCGTCTGCGTTTCTTTCT	AACCGGAAAGATGATGGAAC
daf-16 wt	GTCCTCATTCACTCCCGATT	CTGTCGACCCGTTTGAAGAT
pqm-1mut	GGTTCTCCATTGTGTGCTCA	ACAACCCTCAATTTTGCAGG
pqm-1 wt	ATTCCGTGCCGATAGTTCTT	AACCCCTACCTGATGCAAAA
sod-3	GCTGCAATCTACTGCTCGCACTG	GGCTGATTACAGGTTCCAAATCTGC
eat-2	CGAGAGCCCCAGAATTACAA	ACCTCCCACTATCCCTCCAC
myl-1	GGCTTGCAAGTGTGACTGCAAAAAC	CTTGCAGTCTCCCTTACATCCAGC
fkb-6	AGATCGCGGTGATCAATTCT	AATCCGAGCGAATTGTGAAT
18S	GCCAGCAGCCGCGGTAATTCCAGC	TTGCGAATCTGAGGCACGTAACCT
hsp-25	GTCGTGTTGAGGAGGAGAT	AGAAGAAGTGTTCGAGTAGGC
