# Supplementary material for: Dietary restriction and gonadal signaling differentially regulate post‐development quality control functions in Caenorhabditis elegans
Source: Aging Cell. 2019 Jan 15;18(2):e12891. doi: 10.1111/acel.12891 (PMC6413660; doi:10.1111/acel.12891)
Supplement: Supplementary file 10 [file ACEL-18-e12891-s010.docx]

**Supporting Information**:

**Fig. S1.** **DR modulates the HS response during adulthood.** **(A)** Survival rates of age-synchronized *eat-2(ad453)* animals during adulthood. Animals were subjected to HS (6 h at 37°C) on the indicated day and survival was assayed. **(B)** Survival rates of age-synchronized wt, *eat-2(ad453)* or *eat-2(ad1116)* animals grown at 15°C and subjected to HS (4 h at 34°C) at the indicated ages. **(C)** Expression levels of HS genes. mRNA levels of *hsp-16.2* and *hsp-70* from age-synchronized wt or *eat-2(ad453)* animals untreated or subjected to HS (90 min at 37 °C). Data presented were normalized to day 1 HS-treated wt animals. *P* values were calculated by comparison with day 1 adults of the same strain. (*) denotes P<0.05 and (**) denotes P<0.01.

**Fig. S2.** **Differential requirements for DAF-16.**  **(A)** Nuclear localization of DAF-16. Age-synchronized DAF-16::GFP, *eat-2(ad453);*DAF-16::GFP or *eat-2(ad453);glp-1(e2141);*DAF-16::GFP animals fed *ad libitum* or BD-treated for 24 h were fixed on day 2 of adulthood, imaged and the percentage of animals showing nuclear localization of DAF-16::GFP was scored (>580 cells per treatment, N>3). **(B)** Confocal images of representative DAF-16::GFP-expressing animals (as indicated). Arrows mark nuclear DAF-16::GFP. **(C)** Expression levels of DAF-16-regulated genes. mRNA levels of *mtl-1*, *sod-3* and *hsp-16.2* from age-synchronized wt, *eat-2(ad453)* or *eat-2(ad453)*;*glp-1(e2144)* day 2 adults. *P* values were calculated by comparison with age-matched wt animals. (**) denotes P<0.01.

**Fig S3.** ***jmjd-3.1* is not required for HS survival of BD-treated animals.** Survival rates of age-synchronized *jmjd-3.1(gk384)* animals. Animals were fed *ad libitum* or BD-treated for 24 h, subjected to HS (6 h at 37°C) on day 2 of adulthood and survival was assayed. *P* values were calculated by comparison with day 1 adults of the same strain. (**) denotes P<0.01.

**Fig. S4 Differential requirements for PQM-1. (A-B)** Expression levels of putative DAF-16 and PQM-1 targets. mRNA levels of chaperones, as indicated, from day 2 adult *eat-2(ad453)* or *eat-2(ad453)*;*pqm-1(ok485)* animals subjected to HS (90 min at 37 °C) (A) or untreated (B). Data presented are normalized to those from day 1 *eat-2* animals. *P* values were calculated by comparison with age-matched control animals. (*) denotes P<0.05.

**Fig. S5 GSC-arrested *eat-2* animals require DAF-16 for proteostasis rescue. (A)** Survival rates of age-synchronized *eat-2(ad453)*;*glp-1(e2144)* animals during adulthood. Animals were subjected to HS (6 h at 37°C) on the indicated day and survival was assayed. **(B)** Survival rates of age-synchronized *eat-2(ad453)*;*glp-1(e2144)* animals with or without *daf-16(mus86)* or *pqm-1(ok485)* mutation. Animals were subjected to HS (8h at 37°C) on day 2 of adulthood and survival was assayed. **(C)** Motility of age-synchronized mutant animals as in B. Animal thrashing rates were monitored on day 4 of adulthood. Data were normalized to motility rate of each strain on day 1 of adulthood. **(D)** Survival rates of age-synchronized *eat-2(ad453)*;*glp-1(e2144)* animals fed on the indicated RNAi-expressing bacteria. Animals were subjected to HS (6 h at 37°C) on day 3 of adulthood and survival was assayed. In A, *P* values were calculated by comparison with day 1 adults. In B-D, *P* values were calculated by comparison with age-matched control animals. (**) denotes P<0.01.
